# Post-COVID-19 condition patients’ utilisation of healthcare resources after implementation of an integrated care unit

**DOI:** 10.1186/s12962-025-00667-z

**Published:** 2025-12-02

**Authors:** José Ángel Vicente-Gómez, Martín de Muniategui Climente, Cora Loste, Saúl Barreales, Laura Ricou Ríos, Roger Paredes, Lourdes Mateu, Francesc Lopez Segui

**Affiliations:** 1 Centre for Research in Health and Economics (CRES), Pompeu Fabra University, Catalonia, Spain; 2 Research Group on Innovation, Health Economics and Digital Transformation, Institut Germans Trias i Pujol, Catalonia, Spain; 3 BarcelonaTech, Barcelona, Spain; 4 Department of Infectious Diseases, Hospital Germans Trias i Pujol, Badalona, Catalonia Spain; 5 Fundació Lluita Contra les Infeccions, Badalona, Catalonia Spain; 6 Universitat de Vic – UCC, Vic, Catalonia Spain; 7 REICOP, Madrid, Spain; 8 Direcció d’Atenció Primària Metropolitana Nord, Institut Català de la Salut, Barcelona, Spain; 9 Universitat Autònoma de Barcelona, Catalonia, Spain; 10 IrsiCaixa AIDS Research Institute, Germans Trias i Pujol Research Institute (IGTP), Can Ruti Campus, Badalona, Catalonia Spain; 11 CIBER Infectious Diseases (CIBERINFEC), Institute of Health Carlos III (ISCIII), Madrid, Spain; 12 Center for Global Health and Diseases, Department of Pathology, Case Western Reserve University School of Medicine, Cleveland, OH USA

**Keywords:** COVID-19, Economic evaluation, Healthcare utilisation, Post-COVID-19 condition, Long COVID

## Abstract

**Background:**

The economic effects of post-COVID-19 condition (PCC) remain uncertain despite clearer clinical factors, posing challenges for healthcare professionals. This article investigates the demographic and clinical characteristics of PCC patients and compares their healthcare resource utilization in comparison to a patient cohort representative of the population.

**Methods:**

A retrospective and cohort-comparative observational study was conducted, comparing PCC population before and after diagnosis with a control group. Demographic and clinical variables were analysed to describe the population. Economic analysis was performed to evaluate the resource costs in procedures and primary, secondary and emergency care.

**Results:**

PCC patients (*N* = 341) were older with higher cardiovascular risk factors compared to controls (*N* = 49,078). There were differences in the socio-economic distribution between male and female in the PCC patients. Hypertension and diabetes mellitus type 2 were the most common chronic diseases observed among the case patients. PCC patients were four times as costly as control patients, with increased utilisation of healthcare resources. However, post-diagnosis PCC patients showed a reduction in costs, primarily driven by decreased primary care visits and hospitalisations.

**Conclusions:**

Coordinated care for PCC patients leads to cost reductions and improved resource utilisation. Further research should investigate long-term health outcomes and establish causal relationships between COVID-19 sequelae and healthcare resource utilisation.

**Supplementary Information:**

The online version contains supplementary material available at 10.1186/s12962-025-00667-z.

## Background

Post-COVID-19 condition (PCC) or Long COVID is a long-lasting, poorly understood, and highly disabling post-viral syndrome, which poses enormous healthcare, economic, and socio-political challenges. Between 5% and 10% of individuals who become infected with SARS-CoV-2 will develop a PCC [1–3]. This implies that more than 36 million people in Europe may be affected. This syndrome includes a large number of symptoms that affect the physical, emotional, and social health of individuals, significantly diminishing their quality of life [4, 5]. Despite recent research on potential biomarkers that might guide diagnosis and treatment [6], the pathophysiology of this syndrome remains uncertain, effective treatments are lacking, and validated biomarkers are absent, leading patients with this complex condition to seek multiple consultations with different specialist doctors before receiving a diagnosis [7–11]. Taking all these factors into account, it becomes evident that the responsibility for the care of COVID-19 patients extends beyond the acute phase of the infection – the goal of this specialized integrated care model is to improve patient well-being and optimize healthcare resource use. The Unit, which has been described elsewhere [11], coordinates multidisciplinary treatment by incorporating state-of-the-art knowledge, reducing inefficiencies from fragmented care. The unit follows a structured approach with initial nursing visits, medical assessments, rehabilitation, and follow-ups at different intervals. This underscores the need for interdisciplinary collaboration to ensure comprehensive care in outpatient settings [12]. Therefore, it is imperative for healthcare systems to acknowledge the importance of establishing specialised COVID-19 clinics [13]. The elevated healthcare cost associated with these factors is believed to be potentially minimised by directing visits towards a multidisciplinary Long COVID unit. In this context, the objective of this observational study is to compare the costs of patients with PCC both retrospectively (before and after diagnosis and treatment) and prospectively (in contrast to the control population, in a cohort-comparative setting).

## Methods

### Health economic analysis plan

A health economic analysis plan was not developed before the study was conducted.

### Study population

The design of the study consists of a cohort-comparative population. The case group corresponded to all patients who were admitted to the Long COVID unit between June 2020 and December 2022 (*n* = 341). Patients were diagnosed with the ICD B94.8 code (Sequelae of other specified infectious and parasitic diseases) before the introduction of the U09.9 code for PCC. The only inclusion criteria for the sample under study was for the individual to be over 18 years old, to have a diagnosis of PCC and to reside within the geographic radius of the hospital’s service area, specifically, the North Metropolitan Area of Barcelona (Catalonia, Spain). If the patients fitted the inclusion criteria, they would enter the Long COVID unit and their data would be collected.

The control population was obtained through a matching algorithm from administrative records of the reference. The algorithm is based on a criteria of similarity to the case population by the following variables: sex ratio (approximately 2:1 female-male), age-group (an approximate similarity), and socioeconomic status (based on pharmacy copayments). Having acute COVID or its sequelae was not a criteria for the control population selection, in order to maintain the integrity of the observational study.

The resulting cohort had a size of 49,419 patients: 341 from the case population and 49,078 from the matched case population. Both populations’ use of services was measured from December 2017 to December 2022.

### Setting and location

The study was conducted in the Germans Trias i Pujol Hospital (Spain) and the primary care centres in its area between July 2020 and December 2022. This is the largest monographic Long COVID Unit in Spain. The Long Covid Unit’s objective is to diagnose, assess, and rehabilitate individuals suffering from PCC through multidisciplinary care by physicians, nurses, nutritionists and psychologists [11].

The unit’s guidelines outline a structured approach to patient assessment and follow-up. The process begins with an initial visit during which the nursing team develops a care plan, offers health education, and evaluates the patient’s symptoms. A family doctor then conducts further assessments, such as physical examinations and complementary procedures like chest X-rays, spirometry (SpO2), electrocardiograms, fatigue tests, and functional tests.

Up to six weeks after the initial visit, a first follow-up with the family doctor takes place to assess analytical results and functional test ratings. Based on the severity of the condition and results, patients may be referred to infectious diseases specialists, telematic rehabilitation, or hospitals for further evaluation and treatment. During this period, another visit to the nursing team occurs to provide additional health education and continue the rehabilitation plan.

After six weeks, medical attention is provided either remotely through calls, video calls, or on-site, depending on the patient’s needs and the progress of rehabilitation. At 12 weeks (3 months), tracking visits are scheduled to monitor the patient’s progress and response to treatment, including clinical analysis results, fatigue and functional tests, and overall condition. Based on these evaluations, patients may continue rehabilitation or be referred to higher levels of healthcare for further treatment. At 24 weeks (6 months), a final visit is conducted to conclude the process.

### Comparators

The costs of PCC patients were compared both in a retrospective manner (pre and post diagnosis and treatment) and in a cohort-comparative setting (in contrast with a comparison population). The retrospective analysis covered a symmetrical 12-month period (six months before and after diagnosis), while the prospective analysis in the control population spanned 12 months.

We also retrospectively compare the use of different services in primary and hospital care, procedures, hospitalizations and emergencies. A cohort-comparative comparison in these services would be difficult to measure due to the different orders of magnitude of the sample sizes and the large amount of missing values in the control population.

### Perspective

This study employs a partial patient perspective to estimate the costs produced by post-COVID19 condition patients. The costs of the patient are paid by the hospital, which is then reimbursed by the public insurer (Catalan Health Service), whose financing comes from taxes paid by citizens.

### Time horizon

Regarding the time horizon of the costs, patients were discharged from the Unit after 6 months and subsequently returned to the standard pathway of primary care and specialist visits.

### Discount rate

No discount rate was chosen as the time horizon of the costs was under 1 year.

### Measurement and evaluation of resources and costs

Tables 1 and 2 describe the prices for each form of healthcare resource utilisation. The included resources were primary care visits (in different forms, as specified in the table), outpatient care (first and successive visits of an episode), inpatient care, emergency care and different procedures. Costs were valued per the Catalan Law regulating billable assumptions and concepts and approving the public prices corresponding to the services provided by the Catalan Institute of Health, which acts as a cost catalogue for forms of healthcare resource utilisation [14]. In the results, we present the average number of visits per six months (for the pre-post comparison) and in 12 months (for the cohort comparison).

**Table 1:** Description of costs for healthcare resources

Group	Prices	Number of visits for case population, after diagnosis
Primary care		
General practitioner	50·00 €	2413
Nursery	35·00 €	708
Home visits	65·00 €	6
Emergency care centres	120·00 €	118
Sexual and reproductive care	50·00 €	267
Others (mental health, administrative, extractions)	30·00 €	609
Hospital visits		
First visit	171·00 €	424
Successive and other visits	80·00 €	1411
Hospitalisations		
General	674·00 €	14
Surgery	817·00 €	2
Home hospitalisations	477·00 €	4
Emergencies	248·00 €	124
Procedures	See Table 2	

Costs were priced as per [14], with some services in primary care (Emergency care centres, Sexual and reproductive care, Others) being an average of service costs across different specifications. Procedures were included in Table 2

**Table 2:** Description of costs for each procedure

Code	Name	Price	Code	Name	Price
RA00001	Chest X-ray 1–2 projections	15·00 €	PD00118	Electrocardiogram (ECG or EKG)	21·00 €
PD00642	Liquid-based Gynecological Cytology	15·00 €	RA00561	Shoulder Ultrasound	24·00 €
RA00248	Computed Tomography of the chest without contrast	110·00 €	RA01200	Urinary Tract Ultrasound (Renal - Bladder)	24·00 €
RA00047	Lumbar-sacral spine X-ray 1–2 projections	15·00 €	PD00041	Non-Mydriatic Retinography	93·00 €
PD00008	Electromyography (EMG) of Lower Limbs	100·00 €	PD00069	Colonoscopy (Fibrocolonoscopy) with Sedation	185·00 €
PD00046	Photography of the Eye Fundus (Ophthalmoscopy)	140·00 €	PD00093	Forced Spirometry with Bronchodilator Test	82·00 €
RA00002	Chest X-ray >2 projection	15·00 €	RA00090	Foot X-ray >2 projections	15·00 €
RA00041	Cervical Spine X-ray 1–2 projections	15·00 €	RA00428	Soft Tissue Ultrasound	24·00 €
RA00416	Transthoracic Echocardiogram	67·00 €	RA00020	Face X-ray 1–2 projections	15·00 €
RA00419	Abdominal Ultrasound (Complete)	72·00 €	RA00062	Shoulder X-ray 1–2 projections	15·00 €
RA00429	Transvaginal Ultrasound	36·00 €	RA00038	Orthopantomography (Dental Panoramic Radiography)	25·00 €
IQ44.13-1	Fibrogastroscopy	110·00 €	Others (median price)		40·00 €

Procedures were priced as per [14]. The category “Others” englobes procedures others than the ones listed (the most common used in the Unit population) and is a median price of such procedures

Due to the staggered entry of patients into the program, a fixed time-frame for studying visits was not feasible. Instead, a balanced approach was adopted, setting an individual threshold of six months before and after diagnosis for each patient. Visits were recorded individually in the databases, then aggregated by service and patient. This method was used to calculate averages by age-group, sex, and the entire population.

### Currency, price date, and conversion

In order to monetize different types of services across providers, we valued costs in euros at 2020 prices, following the Catalan healthcare billing law [14]. The reporting of this study follows the Consolidated Health Economic Evaluation Reporting Standards (CHEERS) framework for economic evaluations, as recommended by health economists associations [15].

### Statistical tests

At baseline, demographic and analytical variables were collected for the case population, while the last available value was used for the control population. The median and IQR are used to describe the quantitative variables, while n(%) are used for qualitative variables. The p-value indicates the statistical significance of the difference between the two groups being compared. To test the significance of the differences, we used Wilcoxon, Fisher, Pearson and Kruskal-Wallis tests. Finally, N/A indicates the missing values for each variable. The software R (version 4.2.2) and its packages tidyverse, glmnet, and zoo were used for the data manipulation and statistical analysis [16–19].

### Approach to engagement with patients and others affected by the study

The recruitments of patients in the Unit was used to better understand their needs and to define the personalized care plans. Clinicians and other healthcare professionals involved in the Unit were part of the planning of the study.

### Role of the funding source

Funding for the study was provided through fundraising campaigns organised by the non-profit foundation Fundació Lluita contra les Infeccions, which included “yomecorono.org” and Gala contra les Infeccions, Editions 2021 and 2022. None of the funding sources played a role in the study’s design, data collection, data analysis, interpretation of results, or the writing of the report.

## Results

### Descriptive statistics for the population

We evaluated 49,078 individuals in the control group and 341 in the case group (Table 3, second column). The Case group was older and had higher values for systolic and diastolic blood pressure, body-mass index, coronary risk, cholesterol, ferritin, and glomerular filtration rate compared to the Control group, with no other significant differences in demographic characteristics. Hypertension and diabetes mellitus type 2 were the most common chronic conditions in the Case population, with several other chronic diseases with shared risk factors and comorbidities (cardiovascular disease, obesity, older age) also being overrepresented.

**Table 3:** Statistics of the control and cases populations, and by sex within the case population

	Control (*N* = 49,078)	Case (*N* = 341)	*p*-value	Female (*N* = 224)	Male (*N* = 117)	*p*-value
**Demographics**						
Age (years), median (IQR)	46 (27, 63)	53 (45, 62)	< 0.001	53 (47, 61)	56 (42, 63)	0.7
Age interval, N (%)			< 0.001			0.4
18–55	26,465 (61%)	190 (56%)		131 (58%)	58 (50%)	
56–65	6,133 (14%)	98 (29%)		61 (27%)	38 (32%)	
66–80	7,085 (16%)	46 (13%)		28 (12%)	18 (15%)	
81+	3,720 (8.6%)	7 (2.1%)		4 (1.8%)	3 (2.6%)	
N/A	5,675 (11%)	0 (0%)		0 (0%)	0 (0%)	
≤ 65	32,598 (75%)	288 (85%)		192 (85%)	96 (82%)	
> 65	16,480 (25%)	53 (15%)		32 (15%)	21 (18%)	
Age of diagnosis, median (IQR)	-	-	-	52 (45, 59)	54 (42, 62)	0.6
Male, N (%)	16,824 (34%)	117 (34%)	> 0.9	-	-	-
Pharmacy copayment index, N (%)						0.002
TSI 001	6,603 (13%)	50 (15%)		39 (17%)	11 (9.4%)	
TSI 002	7,479 (15%)	58 (17%)		33 (15%)	25 (21%)	
TSI 003	18,500 (38%)	123 (36%)		92 (41%)	31 (26%)	
TSI 004	9,726 (20%)	99 (29%)		52 (23%)	47 (40%)	
TSI 005	435 (0.9%)	0 (0%)		0 (0%)	0 (0%)	
TSI 006	1,286 (2.6%)	2 (0.6%)		2 (0.9%)	0 (0%)	
N/A	5,049 (10%)	9 (2%)		6 (2.7%)	3 (2.6%)	
**Clinical**,** median (IQR)**						
Systolic pressure (mmHg)	124 (111, 135)	126 (117, 136)	< 0.001	124 (114, 135)	131 (120, 138)	0.002
N/A	20,703 (42%)	31 (9%)		17	14	
Diastolic pressure (mmHg)	75 (68, 82)	78 (71, 86)	< 0.001	78 (71, 85)	80 (74, 87)	0.064
N/A	20,706 (42%)	31 (9%)		17	14	
SpO2 (%)	98 (97, 99)	98 (97, 99)	0.2	98 (97, 99)	98 (96, 98)	0.006
N/A	37,905 (77%)	140 (41%)		88	52	
Height (cm)	156 (127, 164)	164 (159, 169)	< 0.001	160 (157, 164)	172 (167, 177)	< 0.001
N/A	35,289 (72%)	218 (64%)		139	79	
Weight (kg)	68 (56, 82)	76 (65, 86)	< 0.001	69 (62, 81)	84 (78, 94)	< 0.001
N/A	22,960 (47%)	82 (24%)		50	32	
Body-Mass Index	253 (203, 296)	279 (252, 322)	< 0.001	274 (241, 324)	283 (265, 316)	0.2
N/A	24,259 (49%)	112 (33%)		74	38	
Coronary Risk Score	326 (198, 532)	254 (135, 457)	0.001	214 (126, 362)	377 (214, 571)	0.001
N/A	41,002 (84%)	216 (63%)		147	69	
Cholesterol (mg/dL)	196 (167, 226)	211 (187, 238)	< 0.001	211 (187, 239)	210 (184, 230)	0.3
N/A	23,150 (47%)	2 (0%)		1	1	
Ferritin (ng/mL)	50 (22, 111)	80 (36, 170)	< 0.001	55 (28, 106)	172 (101, 286)	< 0.001
N/A	35,144 (72%)	47 (14%)		18	19	
Alkaline phosphatase (IU/L)	80 (65, 102)	74 (61, 88)	< 0.001	74 (61, 88)	74 (62, 84)	0.7
N/A	36,140 (74%)	32 (9%)		17	11	
Glucose (mg/dL)	91 (84, 101)	93 (86, 102)	0.2	90 (84, 100)	95 (88, 106)	< 0.001
N/A	22,389 (46%)	3 (1%)		1	2	
Glomerular filtration rate (mL/min/1.73 m²)	77 (65, 84)	81 (75, 86)	< 0.001	82 (75, 87)	80 (73, 85)	0.12
N/A	39,586 (81%)	286 (84%)		162	71	
**Chronic conditions**,** N (%)**						
Hypertension (I10)	8939 (18%)	90 (26%)				
Diabetes mellitus type 2 without complications (E11.9)	3801 (8%)	40 (12%)				
Other pulmonary embolism (I26.99)	196 (> 1%)	16 (5%)				
Unspecified chronic obstructive pulmonary disease (J44.9)	972 (2%)	12 (4%)				
Unspecified chronic renal disease (N18.9)	2158 (4%)	11 (3%)				
Angina pectoris, unspecified (I20.9)	254 (> 1%)	9 (3%)				
Unspecified atrial fibrillation (I48.91)	1100 (2%)	6 (2%)				
Other unspecified right bundle branch block (I45.0)	656 (1%)	5 (2%)				
Nonrheumatic aortic valve stenosis (I35.1)	201 (> 1%)	5 (2%)				
Mitral valve prolapse (I34.0)	317 (> 1%)	5 (2%)				

P-values for continuous variables come from two-sample t-tests, while those for categorical variables come from Chi-Square tests. The variable TSI indicates the level of copayments in drugs. As Spain uses a progressive copayment system, it serves as a proxy for socioeconomic status. TSI 001 covers people who receive non-contributory pensions (such as people with disabilities); TSI 002 includes pensionists with annual rents under €100,000; TSI 003 covers workers with annual incomes under €18,000; TSI 004 corresponds workers with annual incomes between €18,000 and €100,000; TSI 005 covers both individuals and workers with annual rents or incomes over €100,000; and TSI 006 includes civil servants, military officers and judges under their mutualist insurance, a specific model of health insurance available to aforementioned groups

The third column presents the characteristics of the case population by sex. Sex was included as reported by the physician in the health records. There existed significant differences between male and female users in terms of pharmacy copayment index, systolic pressure, coronary risk, ferritin levels, and glucose levels. The chronic conditions were not broken down by sex given the low N.

### Primary care

We analysed the ratio of visits categorised by age-group and sex (Table 4) from both the retrospective and cohort-comparative perspective. For the retrospective point, the mean number of visits was 2.3 per month before diagnosis and 1.93 after. The reduction was greater on men than on women. All of the population groups perceived a significant reduction in visits ranging from 0.57 to 0.20, except for women over 81 who increased their visits by 0.37. However, this group consists of only 4 women, representing 1.1% of the whole population; this also happens with the 3 men over 81, which saw the biggest reduction in visits (1.90) but barely represent 0.87% of the population. As such, their results are considered not statistically important, neither here nor when we consider other aspects of use of healthcare resources.

**Table 4:** Primary care visits mean broken down by gender and age-group

Sex	Age-group	Pre-post				Cohort-comparative population	
		Before diagnosis	After diagnosis	Pre-post variation	*p*-value	Control visits	Post over control ratio
**All**	**All**	**2·30 (1·79)**	**1·93 (2·00)**	**-19%**	**0.01965**	**0·81 (1·05)**	**2·39**
Female	All	2·38 (1·72)	2·06 (2·11)	-16%	0.06658	0·89 (1·09)	2·30
	18–55	2·46 (1·76)	2·11 (2·35)	-17%	0.198	0·77 (0·97)	2·74
	56–65	2·47 (1·72)	2·27 (1·98)	-9%	0.5503	0·93 (1·04)	2·44
	66–80	1·98 (1·76)	1·42 (1·24)	-39%	0.0384	1·14 (1·13)	1·25
	> 81	1·42 (0·40)	1·79 (1·10)	+ 21%	0.5428	1·40 (1·72)	1·28
Male	All	2·15 (1·92)	1·65 (1·77)	-30%	0.1355	0·63 (0·94)	2·62
	18–55	1·88 (1·64)	1·45 (1·66)	-30%	0.3089	0·44 (0·70)	3·30
	56–65	2·74 (2·10)	2·17 (1·93)	-26%	0.4989	0·75 (1·12)	2·89
	66–80	1·76 (2·20)	1·53 (1·78)	-15%	0.6334	1·05 (1·16)	1·46
	> 81	2·28 (1·95)	0·38 (0·34)	-500%	0.4548	1·27(1·60)	0·30

As for the prospective point, the number of visits made by the control group was significantly smaller than that of the case group, both before and after diagnosis. The overall mean stands at 0.81 visits per month, with higher values for older age-groups and women.

**Fig. 1:**
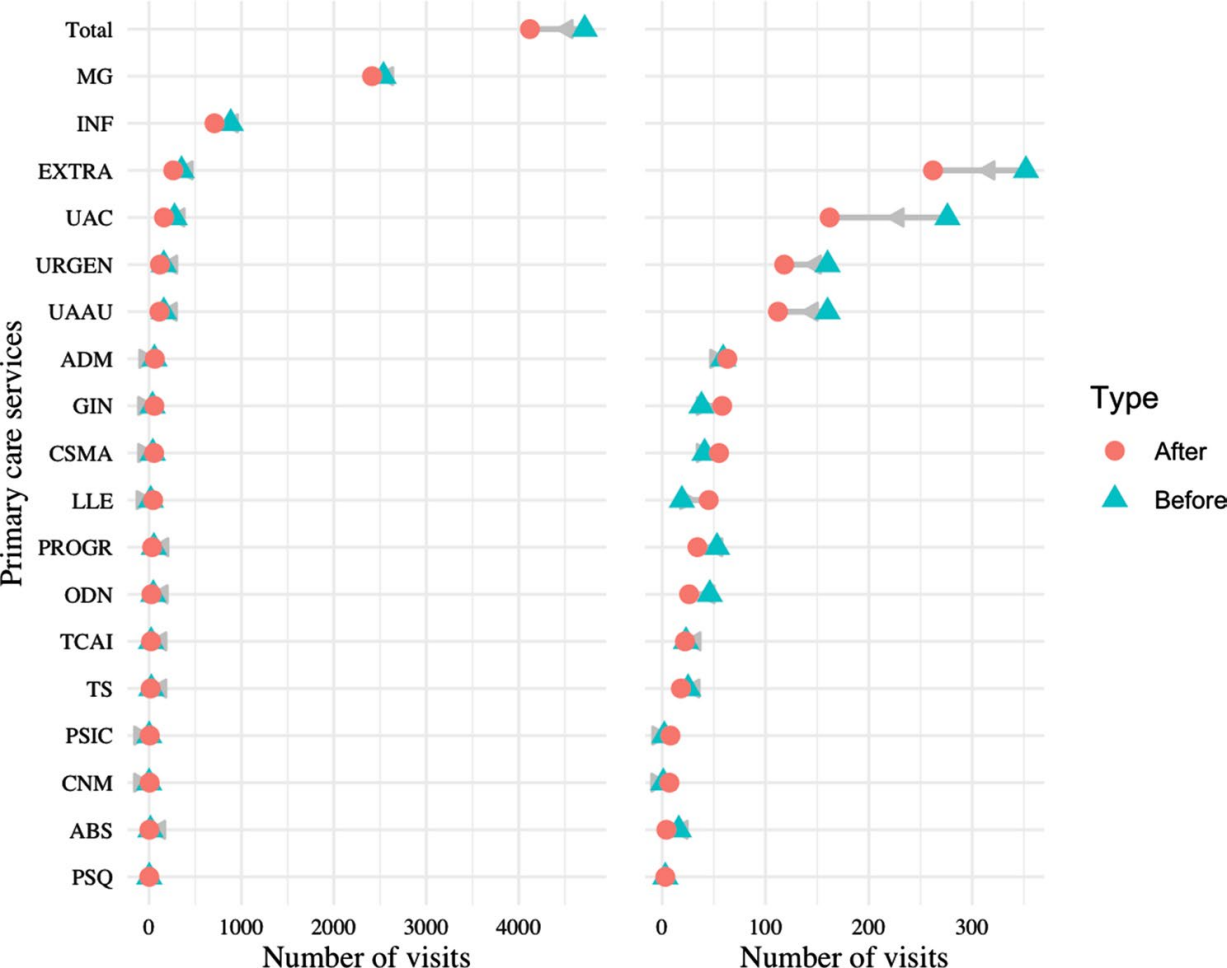
Change in visits in different services of primary care. Dumbbell plot displaying the change in the number of visits before and after diagnosis in different services of primary care. The blue triangle indicates the number of visits before diagnosis, and a red circle indicates the number of visits after diagnosis. The number of visits indicates the number of visits all PCC patients made to each service in the six months prior (and six months after) their PCC diagnosis. The grey arrow indicates the direction of change: a left leaning arrow is a decrease, while a right-leaning arrow is an increase. The plot on the left shows all categories, while the one on the right excludes the first two categories and the total to better visualise services with a smaller amount of visits (notice the change on the scale of the x-axis from 0-4000 to 0-400)

When comparing visits before and after diagnosis patient by patient, individually, in primary care, most patients have under 5 visits both before and after diagnosis, with a great number of them staying under 2.5; the distribution is right-skewed. Figure 1 displays an overall reduction in visits from 4,714 to 4,121 (-12%) as seen in the first category (which totals the rest of services). All services with over 100 visits have a strong reduction in visits.

### Hospital care

We analysed the ratio of visits in hospital care by age-group and sex, again both prospective and retrospective (Table 5). For the retrospective point, the mean number of visits was 0.72 per month before diagnosis and 0.90 after, with an increase of 0.18 visits that was consistent both for men and women. This consistency arises from the fact that most of these visits were under the programme and thus compulsory for all patients. Most groups displayed a consistent increase in visits from 0.18 to 0.24 except for 66–80 year old women, who saw a reduction of 0.13 visits.

**Table 5:** Outpatient hospital visits mean broken down by gender and age-group

Sex	Age-group	Pre-post case population				Cohort-comparative population	
		Before diagnosis	After diagnosis	Pre-post variation	*p*-value	Control population visits	After diagnosis over control ratio
**All**	**All**	**0·72 (0·74)**	**0·90 (0·85)**	**+ 20%**	**0.7144**	**0·15 (0·44)**	**5·93**
Female	All	0·72 (0·75)	0·90 (0·85)	+ 20%	0.4022	0·16 (0·45)	5·68
	18–55	0·60 (0·74)	0·82 (0·87)	+ 27%	0.1183	0·10 (0·30)	8·45
	56–65	0·76 (0·65)	1·00 (0·87)	+ 24%	0.3819	0·22 (0·55)	4·50
	66–80	1·15 (0·84)	1·02 (0·75)	-13%	0.06288	0·32 (0·64)	3·21
	> 81	1·00 (1·01)	0·66 (0·43)	-52%	0.1038	0·28 (0·68)	2·35
Male	All	0·72 (0·73)	0·90 (0·85)	+ 20%	0.07218	0·14 (0·43)	6·49
	18–55	0·65 (0·70)	0·88 (0·90)	+ 26%	0.9454	0·06 (0·20)	14·55
	56–65	0·86 (0·79)	0·93 (0·79)	+ 8%	0.2901	0·20 (0·51)	4·45
	66–80	0·62 (0·66)	0·84 (0·88)	+ 6%	0.05684	0·39 (0·75)	2·14
	> 81	1·06 (1·13)	0·88 (0·69)	-20%	0.4214	0·38 (0·76)	2·30

For the prospective point, the overall mean of the control group stood at 0.15 visits per month. The values were higher for older age-groups, and non-significantly higher for women except in the 65–80 and 81 + age-groups. Nonetheless, all population groups in the control populations made less visits to hospital care than their case population counterparts.

When comparing outpatient visits before and after diagnosis patient by patient, the distribution is right-skewed: most patients have under 3 visits. This time around however, a slightly larger number of points is over the red line, showing an increase of visits.

**Fig. 2:**
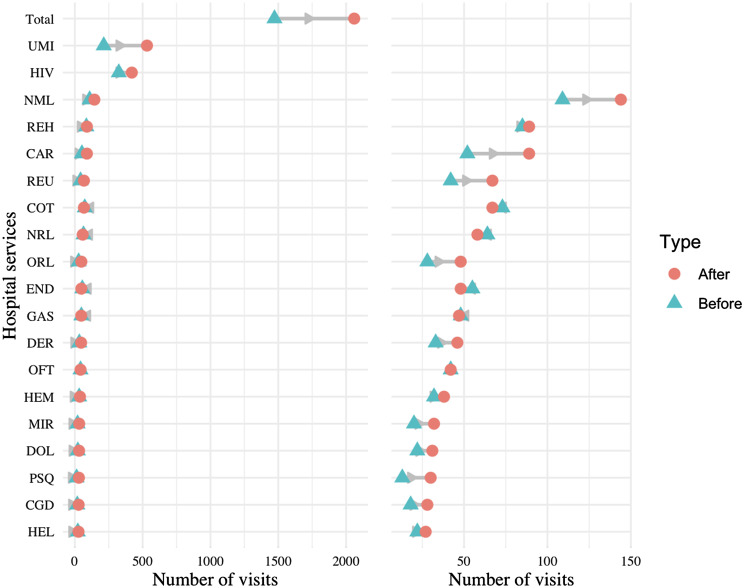
Change in visits in different services of outpatient care. Dumbbell plot displaying the change in the number of visits before and after diagnosis in different services of outpatient care. The blue triangle indicates the number of visits before diagnosis, and a red circle indicates the number of visits after diagnosis. The number of visits indicates the number of visits all PCC patients made to each service in the six months prior (and six months after) their PCC diagnosis. The grey arrow indicates the direction of change: a left leaning arrow is a decrease, while a right-leaning arrow is an increase. The plot on the left shows all categories, while the one on the right excludes the first two categories and the total to better visualise services with a smaller amount of visits (notice the change on the scale of the x-axis from 0-2000 to 0-500)

Figure 2 presents a dumbbell plot similar to Fig. 1, now including hospital services (with more than 5 visits made during the treatment period) instead. There was an overall increase in visits from 1,472 to 1,835 (+ 25%). HIV and infectious diseases (UMI) remained the most-visited services, with pneumology, rehabilitation, cardiology, occupational therapy and rheumatology closely following. Most of the services had an increase in visits, except for occupational therapy, neurology, endocrinology and urology.

### Hospitalizations

When comparing the number of hospitalizations by service in the six months before and after diagnosis, there was a 62% reduction in hospitalizations (from 54 to 20). Most of this decrease was observed in admissions related to infectious diseases and HIV services. All but two services (ophthalmology and gynaecology) experienced a reduction in hospitalizations; when there was an increase, it was of only one visit.

### Hospital emergencies

When comparing the number of hospital emergencies received by each service, there was a reduction of 24%, from 164 to 124. As expected, the general emergency service received 60% of the visits. Most services did not experience a significant change, except for the general emergency service, obstetrics (which experienced a slight increase) and orthopaedics/traumatology (which experienced a slight reduction).

### Procedures

Comparing the six months before diagnosis with the six months after diagnosis, there was an overall reduction of 32% in the total number of procedures, from 320 to 128. The most common order was that of R00001, chest X-rays, which accounted for 29% of the total orders before diagnosis and 20% after. Second place went to PD00642, liquid-based cytology, which saw the strongest increase at 183%. Behind were chest CT (RA00248) and spine CT (RA00047) scans.

### Monetization

Finally, Table 6 acts as a monetization of the healthcare resources described. These costs are presented for an individual patient in a single month.

**Table 6:** Monetization of different types of healthcare resources, standardised by patient per month

Group	Pre-	Post-	Control	Pre-post variation	Post over control ratio
*Primary care*	*103·70 €*	*86·92 €*	*34·25 €*	*-16%*	*2·54*
General Medicine	61·75 €	55·82 €	14·39 €	-10%	3·88
Nursery	14·95 €	11·70 €	7·98 €	-22%	1·47
Home visits	0·70 €	0·32 €	1·20 €	-55%	0·26
Emergency primary care	8·97 €	4·69 €	1·90 €	-48%	2·46
Sexual and reproductive care	1·59 €	3·10 €	1·85 €	95%	1·68
Others	15·73 €	11·29 €	6·92 €	-28%	1·63
*Hospital care*	*70·81 €*	*90·61 €*	*14·70 €*	*28%*	*6·16*
First visit	24·91 €	35·44 €	4·70 €	42%	7·54
Successive visits and others	45·90 €	55·17 €	10·00 €	20%	5·52
*Hospitalizations*	*17·66 €*	*6·34 €*	*4·52 €*	*-64%*	*1·40*
General	10·54 €	4·61 €	2·82 €	-56%	1·64
Surgery	4·79 €	0·80 €	1·58 €	-83%	0·51
Home hospitalizations	2·33 €	0·93 €	0·12 €	-60%	7·48
*Procedures*	*6·82 €*	*4·97 €*	*1·70 €*	*-27%*	*2·93*
*Emergency care*	*19·88 €*	*15·03 €*	*5·40 €*	*-24%*	*2·78*
***Total***	***218·87 €***	***203·87 €***	***56·05 €***	***-7%***	***3·64***

Primary care is disaggregated into several groups by service; hospital care, by whether the visit is the first one or successive/others; hospitalizations, by its character in general, surgery or home hospitalizations. Neither emergencies nor procedures are disaggregated. Finally, it presents the total cost of all patients in each group (341 for both pre- and post-diagnosis, 49,078 for control) for six months; then for an individual patient at six months; and finally for an individual patient in a single month.

Comparing the case and control populations, the former is 3.64-fold as expensive against the post-diagnosis population. Comparing pre- and post-diagnosis populations, there is a reduction in primary care costs (16%), hospitalizations (64%), procedures (27%) and emergency care (24%), while there is an increase in hospital care costs (27%), mirroring the results provided earlier in this section.

## Discussion

While survey-based reports of PCC recovery have found high (35%) cure rates [4, 7] and the PHOSP-COVID cohort had a 29% recovery rate after one year [20], the study conducted at this Long COVID Unit found that only 7.6% of the total PCC patients experienced recovery [11]. Regardless of the exact cure rates, Long COVID might have a global impact on clinical and public health as a long-lasting chronic condition, as at least 5% of those afflicted with COVID-19 are expected to suffer from PCC [1–3]. As such, it is essential to evaluate the rising costs these patients might bear on the system and to adopt policies to contain such costs.

In this 1-year retrospective economic evaluation we found that post-treatment PCC patients are around 7% less costly than pre-treatment patients in terms of use of healthcare resources. While there is a strong increase in hospital care costs, the rest of services have a reduction in costs, notably hospitalizations and primary care. A possible interpretation is that by redirecting visits towards hospital care, an effective, coordinated, integrated treatment can be provided to the patients and reduce their need to access primary care and the overuse of hospitalizations, emergency services and procedures.

Worryingly, PCC patients, no matter whether before or after treatment nor between sex or age-groups, are nearly four times as costly than the control patients. They are high frequency users, and present important differences in their relationship to the healthcare system and processes. Their use of resources, both before and after, greatly surpasses the baremos established by previous systematic reviews [21–23].

The case population had overrepresented chronic conditions, most of which share certain risk factors or comorbidities, such as cardiovascular disease, obesity, and older age, which are known to increase the risk of PCC [4–7, 11, 20]. For example, pulmonary embolism and chronic obstructive pulmonary disease are both associated with impaired lung function and decreased oxygenation, which may contribute to the persistence of COVID-19 symptoms. Similarly, angina pectoris, unspecified atrial fibrillation, mitral valve prolapse, right bundle branch blocks, and nonrheumatic aortic valve stenosis are all cardiovascular conditions that may increase the risk of adverse outcomes in COVID-19 patients.

Consistent with previous evaluations [24–26], we found that PCC patients bore more costs on public healthcare compared to those without symptoms, with the population being older, of female sex and with comorbidities. These studies emphasise the financial implications PCC patients have on the healthcare system and the substantial increase in healthcare utilisation and direct medical costs. Other studies have instead focused on production loss and employment subsidies at a national level [27], specifically focusing on labour supply reduction as a direct result of PCC-induced disabilities [28–30], extending the literature on the financial implications of Long COVID beyond medical costs.

Our study, in comparison, aims to cover the reduction in costs associated with tailored, coordinated care. If treatment is structured and redirects visits from primary care, there should be less visits to primary care and other services, as most of these are driven by the patients’ desperation to obtain medical care [11]. This situation is exacerbated by Spain’s NHS system, where care is free at point of use and demand is only managed through waiting times, which might worsen the patients’ desperation. In this context, cost reduction should not be considered an objective in itself, but rather a secondary effect of tailored treatment. The Unit looks after PCC patients in a more conscious and specific way, and consequently it also avoids potentially irrelevant expenses. As we have presented, a coordinated treatment plan increases outpatient care costs, but reduces utilisation of all other forms of care, and overall reduces the patients’ costs.

Most of our limitations overlap with those of observational studies, notably: (1) the lack of randomization, which forbids us from establishing the causal relationship that the entrance in the care unit leads to less use of healthcare resources, and (2) the constrained reproducibility, as the study is conducted in a natural setting and not in an experimental one. Furthermore, as the case cohort was created early in the pandemic (June 2020), PCC was not fully recognized and there might have been a selection bias favouring severe cases that might make more use of healthcare resources.

Two limitations arose in the data selection. First, that having suffered from COVID or sequelae was not a criteria when selecting the control population, which results in a potential bias of comparing ‘healthy’ citizens to ‘ill’ post-COVID-19 patients. Nonetheless, using an experimental design for treatment would have raised ethical concerns. Second, that some of the visits and hospitalizations during the six months previous to diagnosis in the post-COVID-19 population could be due to COVID-19. These two limitations are in line with those of observational studies in comparison with experimental studies such as RCTs, where these variables could have been controlled.

A final limitation is the lack of measurement of health outcomes, which could have been captured using metrics such as QALYs or DALYs. This omission restricted the evaluation to a cost analysis rather than a full cost-outcome economic evaluation.

The lack of data regarding the prevalence of PCC and other COVID-19 sequelae might underestimate the estimations made regarding the burden these patients bear over the healthcare system. This study provides information regarding their use of healthcare resources and their classification as frequent attenders of care services. Further developments in PCC diagnosis and treatment should center the process around primary care and family doctors to further reduce costs, as early diagnosis of symptoms could improve recovery rates and redirect visits towards a specialized unit. As COVID-19 becomes routinely managed worldwide, its sequelae will have to be tackled in the context of the challenge of chronicity in societies that are undergoing or have undergone epidemiological and demographic transitions. To ensure the sustainability of welfare and healthcare systems, novel forms of care management such as disease management programmes and integrated/coordinated care forms will have to be implemented to depressurize health services off these frequent attenders.

## Conclusions

In summary, this study suggests a coordinated form of care for PCC patients is associated with a reduction in costs. Considering the reduction in primary care visits, hospitalizations and use of emergency services, integrated care outperforms usual management for selected PCC patients. The implementation and management of these forms of care will have to be spread in the future to tackle the growing costs and overall burden of chronic patients in European healthcare systems.

## Supplementary Information

Below is the link to the electronic supplementary material.

Supplementary Material 1

Supplementary Material 2

Supplementary Material 3

## Data Availability

No datasets were generated or analysed during the current study.

## Abbreviations

PCC: Post-COVID-19 Condition
CHEERS: Consolidated Health Economic Evaluation Reporting Standards
