# Supplementary material for: Post-COVID-19 condition patients’ utilisation of healthcare resources after implementation of an integrated care unit
Source: Cost Eff Resour Alloc. 2025 Dec 2;23:72. doi: 10.1186/s12962-025-00667-z (PMC12681129; doi:10.1186/s12962-025-00667-z)
Supplement: Supplementary file 2 — Supplementary Material 2 [file 12962_2025_667_MOESM2_ESM.docx]

Primary care:

- MG: Family Medicine
- INF: Nursing
- EXTRA: Extractions (Blood tests and other clinical tests)
- UAC: Citizen Assistance Unit
- URGEN: Emergencies
- UAAU: Administrative Citizen Assistance
- ADM: Administrative Tasks
- GIN: Gynecology
- CSMA: Mental Health Center for Adults
- LLE: Midwifery
- PROGR: Continuous Nursing Care
- ODN: Dentistry
- TCAI: Nursing Assistant Technician
- TS: Social Worker

Hospital care:

- UMI (Infectious Diseases Service)
- HIV (HIV Unit)
- NML (Pulmonology)
- REH (Physical Medicine and Rehabilitation)
- CAR (Cardiology)
- REU (Rheumatology)
- COT (Oral and Maxillofacial Surgery)
- NRL (Neurology)
- ORL (Otorhinolaryngology)
- END (Endocrinology and Nutrition)
- GAS (Gastroenterology - Digestive System)
- DER (Medical-Surgical Dermatology and Venereology)
- OFT (Ophthalmology)
- HEM (Clinical Hematology)
- MIR (Internal Medicine)
- DOL (Pain Unit)
- PSQ (Psychiatry)
- CGD (General and Digestive Surgery)
- HEL (Hematology Laboratory)
