# Supplementary material for: Post-COVID-19 condition patients’ utilisation of healthcare resources after implementation of an integrated care unit
Source: Cost Eff Resour Alloc. 2025 Dec 2;23:72. doi: 10.1186/s12962-025-00667-z (PMC12681129; doi:10.1186/s12962-025-00667-z)
Supplement: Supplementary file 3 — Supplementary Material 3 [file 12962_2025_667_MOESM3_ESM.docx]

There are 272 different chronic disease codes for the control population and 55 codes for the treatment population. This table describes the most common diseases in the treatment (≥ 5 cases) and control (≥ 350 cases) populations:

| **Code** | **Disease Name** | **Description** |
| --- | --- | --- |
| C01-I10 | Essential (primary) hypertension | A condition where the blood pressure in the arteries is consistently elevated. |
| C01-E11.9 | Type 2 diabetes mellitus without complications | A metabolic disorder characterized by high blood sugar levels due to insulin resistance or insufficient insulin production. |
| C01-I26.99 | Other pulmonary embolism | A blockage in the pulmonary artery or its branches, typically caused by a blood clot that has traveled from elsewhere in the body. |
| C01-J44.9 | Chronic obstructive pulmonary disease, unspecified | A group of lung diseases that cause airflow obstruction and breathing difficulties, such as emphysema and chronic bronchitis. |
| C01-N18.9 | Chronic kidney disease, unspecified | A progressive condition in which the kidneys gradually lose function over time. |
| C01-I20.9 | Angina pectoris, unspecified | Chest pain or discomfort caused by reduced blood flow to the heart muscle. |
| C01-I48.91 | Unspecified atrial fibrillation | A type of irregular heartbeat that can increase the risk of stroke and other complications. |
| C01-I34.0 | Mitral valve prolapse | A condition where the valve between the heart’s left upper and lower chambers doesn’t close properly, allowing blood to leak backward. |
| C01-I45.0 | Other and unspecified right bundle branch block | A delay or blockage in the electrical signal that travels through the right bundle branch of the heart’s electrical system. |
| C01-I35.1 | Nonrheumatic aortic valve stenosis | A condition where the opening of the heart’s aortic valve is narrowed, making it harder for blood to flow from the heart to the rest of the body. |
| C01-I73.00 | Raynaud’s syndrome without gangrene | A condition where the blood vessels in the fingers and toes spasm and narrow in response to cold or stress, causing pain and discoloration. |
| C01-I63.9 | Cerebral infarction, unspecified | A type of stroke that occurs when blood flow to the brain is blocked, resulting in brain cell damage and neurological symptoms. |
| C01-I25.9 | Chronic ischemic heart disease, unspecified | A condition where the heart’s blood vessels are narrowed or blocked, reducing blood flow and oxygen supply to the heart muscle. |
| C01-I73.9 | Peripheral vascular disease, unspecified | A condition where the blood vessels outside the heart and brain, especially in the legs and feet, become narrow or blocked. |
| C01-E11.319 | Type 2 diabetes mellitus with unspecified diabetic retinopathy | A complication of diabetes that affects the eyes and can lead to vision loss or blindness if left untreated. |
